# Mutations and polymorphisms of genes moderate increase in gastric cancer risk

**DOI:** 10.1186/1897-4287-10-S3-A22

**Published:** 2012-04-20

**Authors:** Urszula Teodorczyk, Cezary Cybulski, Anna Jakubowska, Teresa Starzyńska, Małgorzata Ławniczak, Paweł Domagała, Katarzyna Ferenc, Krzysztof Marlicz, Zbigniew Banaszkiewicz, Rafał Wiśniowski, Jan Lubiński

**Affiliations:** 1 International Hereditary Cancer Center, Department of Genetics and Pathology, Szczecin, Poland; 2 Clinic of Gastroenterology, Pomeranian Medical University, Szczecin, Poland; 3 Clinic of General Surgery, University Hospital, Bydgoszcz, Poland; 4 Beskidian Center of Oncology, Bielsko Biała, Poland

## 

Results of studies performed in our center point at DNA changes of many cancer (mostly moderate) susceptibility genes. Association of "weak" mutations and polymorphism of many genes and additional influence of environmental factors can significantly increase the risk of cancer development.

The preliminary studies performed in our department indicate, that increased risk of gastric cancer may be related to mutation in NOD2 and CHK2 genes predisposing to cancers of many organs.

The CHEK2 gene encodes checkpoint kinase 2 (Chk2), it acts as a tumor suppressor whose functions are central to the induction of cell cycle arrest and apoptosis by DNA damage.

Results of studies performed in our center indicate that the frequency of the IVS2+1G→A alteration was significantly increased in a group of 658 consecutive patients, especially in those under 50 years of age (OR=5.332, p=0.0174). I157T was over-represented in the group of familial gastric cancer patients, particularly in patients diagnosed at less than 50 years of age (OR=3.687, p=0.0011) and females. The specific group of intestinal and mixed histopathological subtype occurs more frequently (OR=1.671) in consecutive carriers of I157T mutation.

Recently, the NOD2/CARD15, gene located on chromosome 16q12, has been associated with Crohn’s disease. It is believed to play a role both in intracellular recognition of lipopolysaccharides (LPS) typical of Gram negative bacteria and subsequent activation of NFkB. 3020insC NOD2 mutation causes an increased risk of developing chronic inflammatory diseases of the gastrointestinal tract and colorectal cancer in their course. In light of recent studies, in fact 3020insC mutation predisposes to cancers of many organs, including breast, ovarian, lung. Studies conducted in our center in the group of 103 patients with a family history of gastric cancer, have shown that NOD2 3020insC carriers in persons over 50 y of age more than doubled (OR=2.479, p=0.022) and among women almost 3-fold increased risk. In addition, NOD2 R702W alteration occurred almost three times more frequently (OR=2.816, p=0.0121) in the group of 241 consecutive patients with gastric cancer diagnosed before 50 years of age and over 2-times frequently (OR=2.268, p=0.0371) in gastric cancer selected histopathologically as intestinal type according to Lauren.

There are a large number of reports describing the relationship between changes in the gene TP53, particularly polimomorfizm Arg72Pro, and risk of gastric cancer. Many researchers have found links between gene polymorphism of Interleukin-1, and the risk of stomach cancer. Gene polymorphism of angiotensin converting enzyme ACE, was recently linked with the pathogenesis and development of human cancers. Research Ebert MP, et al. conducted in a group of 88 patients shows that the insertion / deletion ACE gene may be associated with the development of early gastric cancer in the German population. In the Japanese population genetically distant, however, found no relationship between gastric cancer and ACE gene polymorphism). Sentences on the ACE polymorphism and risk of stomach cancer researchers are divided among different centers.

XRCC1 gene is part of composite protein complex containing the ß-polymerase DNA ligase I and PARP (polymerase poly (ADP-ribose). This multi-unit complex is involved in DNA repair pathway. T. Poplawski et al. indicate that the variant Gln / Gln XRCC1 gene polymorphism Arg399Gly occurred more frequently in patients with gastric cancer and family burden (OR=2.04, 95% CI=1,27-3,29) and the results obtained from studies in the group of 152 patients with a family history of gastric cancer suggest that gene XRCC1 399Gln allele may be considered a marker of gastric cancer.

In terms of predisposition to gastric cancer has also been studied recently LAPTM4B gene associated with a genetic predisposition to liver cancer, having LAPTM4B alleles * 1 and LAPTM4B * 2. The risk of stomach cancer was increased 1.819 times in patients with genotype * 1 / 2 (CI=1.273-2.601) and 2.387 times in patients with genotype * 2 / 2 LAPTM4B (CI=1.195-4.767) compared with genotype * 1 / first. These studies indicate that allele * 2 LAPTM4B may be one of the genetic factors of gastric cancer.

Further studies of newly described mutation moderately increased risk of stomach cancer may allow to estimate the real risk of gastric cancer associated with these mutations in the Polish population.

